# Oncometabolites drive tumorigenesis by enhancing protein acylation: from chromosomal remodelling to nonhistone modification

**DOI:** 10.1186/s13046-022-02338-w

**Published:** 2022-04-15

**Authors:** Yidian Fu, Jie Yu, Fang Li, Shengfang Ge

**Affiliations:** Department of Ophthalmology, Shanghai Key Laboratory of Orbital Diseases and Ocular Oncology, Ninth People’s Hospital, Shanghai JiaoTong University School of Medicine, Shanghai, 200025 P.R. China

**Keywords:** Metabolites, Tumour, Lysine acylation, Epigenetic modification

## Abstract

Metabolites are intermediate products of cellular metabolism catalysed by various enzymes. Metabolic remodelling, as a biochemical fingerprint of cancer cells, causes abnormal metabolite accumulation. These metabolites mainly generate energy or serve as signal transduction mediators via noncovalent interactions. After the development of highly sensitive mass spectrometry technology, various metabolites were shown to covalently modify proteins via forms of lysine acylation, including lysine acetylation, crotonylation, lactylation, succinylation, propionylation, butyrylation, malonylation, glutarylation, 2-hydroxyisobutyrylation and β-hydroxybutyrylation. These modifications can regulate gene expression and intracellular signalling pathways, highlighting the extensive roles of metabolites. Lysine acetylation is not discussed in detail in this review since it has been broadly investigated. We focus on the nine aforementioned novel lysine acylations beyond acetylation, which can be classified into two categories: histone acylations and nonhistone acylations. We summarize the characteristics and common functions of these acylation types and, most importantly, provide a glimpse into their fine-tuned control of tumorigenesis and potential value in tumour diagnosis, monitoring and therapy.

## Background

Metabolites are intermediate products of cellular metabolism catalysed by various enzymes inherent to cells, and their interactions within a biological system are collectively known as the metabolome [1]. Metabolic remodelling, as a well-recognized hallmark of malignancy, generates sufficient energy to fuel the exponential growth and proliferation of tumour cells [2–4]. Tumour-inducing metabolic disturbances result in aberrant accumulation of metabolites, also termed oncometabolites [5]. In addition to being involved in metabolic pathways and energy supply, metabolites trigger oncogenic signalling via noncovalent interactions with macromolecules, mainly involved in competitive inhibition or allostery [1]. In one noteworthy example, the signalling molecule succinate binds to and activates succinate receptor 1 (SUCNR1) to promote tumour cell proliferation and metastasis [6–8]. Importantly, metabolites have been shown to possess nonmetabolic functions because of direct protein modifications, which are distinguished from other modifications, such as ubiquitination and sumoylation. These modifications greatly enrich the level of protein modification and expand the functions of metabolites.

Chemical modifications of proteins are also referred to as posttranslational modifications (PTMs). DNA instructs the synthesis of proteins from only 20 primary amino acids; however, PTMs increase the functional diversity of proteins and the proteome [9, 10]. These modifications, which include lysine acylation, can regulate protein activity, turnover, localization, and dynamic interactions with cellular molecules, such as other proteins, nucleic acids, lipids and cofactors [11, 12]. For instance, the succinylation of glutaminase (GLS) at lysine residue 164 (K164) marks GLS for ubiquitination at residue K158 and facilitates its subsequent degradation [13]. Additionally, the enzymatic activity of glutaryl-CoA dehydrogenase (GCDH) is suppressed by glutarylation, causing the lysine oxidation level to decrease [14]. These modifications in turn can influence almost all aspects of normal cell biology and pathogenesis. Notably, PTMs are, beyond all doubt, of supreme importance in tumorigenesis.

**Fig. 1:**
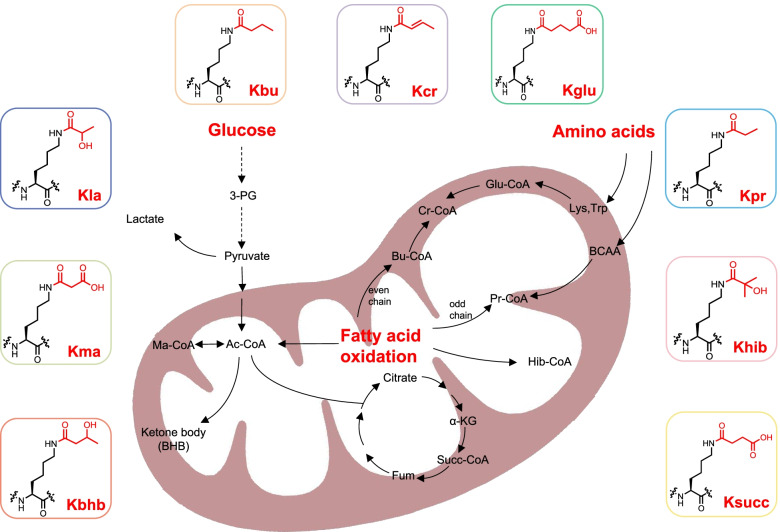
The metabolic association of lysine acylation. Three major metabolic resources(glucose, fatty acid and amino acids) generate abundant intermediate product within cells, such as lactate, Succ-CoA, Ac-CoA and BHB, which provide acyl-groups to covalently modify proteins. Corresponding metabolites of Kcr, Kbu, Kpr, Kglu, Khib mainly emanate from fatty acid oxidation and amino acid metabolism whereas those of Kla, Kma, Kbhb and Ksucc are derived from glucose metabolism

In the past few decades, lysine acetylation (Kac), an abundant, reversible, and highly regulated PTM, has irrefutably become a ‘hot spot’ in the field of epigenetic modification and is precisely modulated by proteins involved in its deposition (writers), binding (readers) and removal (erasers) [15–18]. Owing to the rapid development and application of mass spectrometry technology [9, 19], a large panel of novel lysine acylation types have successively been identified, including lysine crotonylation (Kcr) [20], lactylation (Kla) [21], succinylation (Ksucc) [22], propionylation (Kpr) [23], butyrylation (Kbu) [23], malonylation (Kma) [24], glutarylation (Kglu) [25], 2-hydroxyisobutyrylation (Khib) [26] and β-hydroxybutyrylation (Kbhb) [27]. They stem from major metabolites including glucose, amino acids and fatty acids (Fig. 1). These modifications can be classified into two main categories: histone acylation and nonhistone acylation. Histone acylation mainly affects the epigenome via transcriptional regulation of gene expression, and nonhistone acylation mainly modulates protein functions such as protein stability, activity and localization, as well as protein-protein interactions. In this review, we comprehensively introduce the nine novel metabolite-induced cognate lysine acylations beyond acetylation and elucidate their dramatic roles in metabolomic-epigenetic and metabolomic-proteomic signalling in tumours (Table 1).

**Table 1:** Characteristics and functions of lysine acylations

Acylations	Writers	Readers	Erasers	Metabolites sources	Functions	Ref
Kcr	P300/CBP; GNATs(PCAF);MYSTs(MOF,KAT6A, HBO1, Tip60)	YEATS domain(AF9;Taf 14;YEATS2;ENL) DPF domain(DPF2, MOZ, TAF1)	HDAC1–3; SIRT1–3	Crotonyl-CoA; crotonate	Activate transcription	[28]
					Regulate spermatogenesis	[20, 29, 30]
					Regulate DNA damage response	[31, 32]
					Ensures accurate spindle positioning	[33]
					Protect renal function	[34]
					Ameliorate depression	[35]
					Reactivate latent HIV	[36]
					Facilitate telomere maintenance and differentiation of stem cells	[37, 38]
Kla	P300	NA	NA	Lactyl-CoA; LGSH	Activate transcription	[39]
					Facilitate cell reprogramming	[39]
					Foster lung fibrosis	[40]
					Promote M1-M2 polarization	[21, 41]
Ksucc	GNATs(GCN5,HAT1);CPT1A; KGDHC	YEATS domain(GAS41)	SIRT5; SIRT7	Succinyl-CoA; Succinate	Activate transcription	[42, 43]
					Impair mitochondria respiration, mitophagy and metabolic flexibility	[44]
					Deter neuro filaments aggression in AD	[45–48]
					Exacerbate hypertrophic cardiomyopathy and ischemia-perfusion injury	[49–53]
Kpr	P300/CBP; GNATs(GCN5,PCAF); MYSTs(MOF, MOZ, HBO1)	Bromodomain(BRD4,BRPF1); YEATS domain	SIRT1–3	Propionyl-CoA; propionate	Activate transcription	[54, 55]
					Facilitate protein aggregation in neurodegenerative diseases	[56]
Kbu	P300/CBP; HBO1	Bromodomain(BRD4,BRD9,CECR2,TAF1,Brdt,BRPF1);YEATS domain	SIRT1–3	Butyryl-CoA; butyrate	Activate transcription	[54, 57]
					Promote spermatogenesis	[58]
Kma	NA	NA	SIRT2; SIRT5	Malonyl-CoA; malonate	Facilitate inflammation	[59]
					Involve in malonic aciduria, diabetes-induced NTDs and OA	[60–63]
Kglu	P300; GNATS(GCN5)	NA	SIRT5; SIRT7	Glutaryl-CoA; Glutarate	Activate transcription	[25, 64]
					Destabilize nucleosome structure	[64]
					Involve in GA1 disease	[65]
					Maintain sperm motility	[66]
Khib	P300; MYSTs(ESa1p,Tip60)	NA	HDAC1–3; Rpd3p;Hos3p;CobB	2-hydroxyisobutyryl-CoA;2-hydroxyisobutyrate	Activate transcription	[26]
					Promote spermatogenesis	[26]
Kbhb	P300	NA	SIRT3;HDAC1–3	β-hydroxybutyryl-CoA;β-hydroxybutyrate	Activate transcription	[27]
					Promote memory development of CD8^+^ Tmem cells	[67]
					Antagonize glomerulosclerosis induced by diabetes	[68]
					Alleviate depressive behaviors	[69]

### Crotonylation

#### Characteristics and functions of Kcr

Scientists discovered that Kcr, a novel histone mark, functions distinctively from Kac [20]. Notably, Kcr can be modulated enzymatically by P300/CBP with sodium crotonate serving as the substrate [70] and metabolically on the basis of the concentration of the intermediate metabolite crotonyl-CoA, which increases during mitochondrial or peroxisomal fatty acid oxidation, as well as lysine and tryptophan metabolism [28]. Later, PCAF, MOF, KAT6A, HBO1 and Tip60 were identified as catalysts of Kcr [33, 71–74], and the opposite reaction is mediated by the histone deacetylases HDAC3 [75] and SIRT1–3 [76], with SIRT3 being the major decrotonylase in living cells [77]. Notably, a recent study asserted that class I HDACs are the principal histone decrotonylases [78]. Interestingly, Kcr of HDAC1 itself undermines its deacetylation activity on histone substrates, hinting at the intricacy of PTM regulation [70]. The AF9 YEATS domain has been reported to be a ‘reader’ of histones modified by Kcr with a binding preference for crotonylated lysine sites over lysine acetylation sites in histone H3 [79, 80]. In addition to AF9, the YEATS domains of Taf14 and YEATS2, the double PHD finger (DPF) domains of MOZ and DPF2, and the second bromodomain of TAF1 are capable of binding crotonyl marks [81–84].

P300-mediated histone Kcr has been reported to stimulate gene transcription to a greater degree than histone Kac [28]. However, histone decrotonylation leads to global transcriptional repression and impairs promoter recruitment of its reader proteins in mammalian cells [78]. Histone Kcr marks enhancers and transcriptional start sites (TSSs) of active sex chromosome-linked genes in postmeiotic male germ cells by conferring resistance to transcriptional repressors, which enables successful escape of transcriptional meiotic sex chromosome inactivation (MSCI) [85, 86] and regulates the differentiation of male germ cells [20, 29]. Chromodomain Y-like protein CDYL acts as a crotonyl-CoA hydratase to reduce histone Kcr levels and regulate spermatogenesis [30]. According to previous findings that histone Kcr participates in the DNA damage response [31] and CDYL promotes homology-directed repair (HDR) [87, 88], scientists further revealed a key role of CDYL-regulated RPA1 crotonylation in the HDR process [32]. In addition, Kcr of EB1, a core and scaffold microtubule plus-end tracking protein, ensures accurate spindle positioning in mitosis [33]. Kcr-AF9 YEATS interactions are promoted by crotonate pretreatment, positively regulating gene expression in the inflammatory response [80]. In acute kidney injury (AKI), histone Kcr counters inflammation and mitochondrial stress to protect renal function via transcriptional regulation, demonstrating its potential therapeutic value in renal diseases [34]. Low levels of histone Kcr in the medial prefrontal cortex promote stress-induced depressive behaviours in rodents by regulating gene transcription, thus laying a foundation for innovative epigenetic treatment in depression [35]. In addition, histone Kcr can be targeted to eradicate HIV since the previously latent HIV is reactivated upon hypercrotonylation induced by crotonyl-CoA production [36]. Additionally, histone Kcr has been demonstrated to facilitate telomere maintenance [37] and the differentiation [38] of stem cells.

#### Role of Kcr in cancer

Kcr is considerably reduced in prostate tumours and has a positive correlation with tumour grade. Furthermore, in-depth investigations have documented that histone hypocrotonylation induced by bromodomain-containing protein 4 (BRD4) inhibitors can abrogate cell proliferation and migration, thus showing the potential value of histone hypocrotonylation for therapeutic intervention in prostate cancer [89]. Histone Kcr is also abundant in gut cells, especially H3K18cr, which is associated with increased expression of genes involved in cancer and regulation of cell cycle progression [90]. Moreover, HDAC2, which is elevated in colon cancer and related to tumorigenesis, exhibits decrotonylase activity and thus downregulates histone Kcr levels, meaning that Kcr may participate in cancer suppression [90–92]. Interestingly, the YEATS domain, which has a higher affinity for Kcr sites than other acylation sites [79, 81, 82], plays an important role in leukaemia [93, 94], and inhibition of this domain can attenuate oncogene transcription in leukaemia [94]. Hence, we surmise that the strong epigenetic impact of the YEATS domain on leukaemia may be partially due to its interaction with crotonylated histone. In addition, K420cr of enolase 1 (ENO1) increases its activity, which fosters the proliferation, migration, and invasion of colorectal cancer cells [95]. Wan et al. found that global Kcr levels are reduced in liver, stomach, and kidney carcinomas but increased in thyroid, oesophageal, pancreatic and lung carcinomas. In particular, increased Kcr levels hamper hepatoma cell motility and proliferation [96]. The highly upregulated proteins with Kcr marks in small cell lung cancer are crucial regulators of tumour metastasis or the tumour microenvironment or are involved in wound healing, functions that are all hallmarks of cancer [97–99]. Additionally, since defective double-strand break (DSB) repair leads to genomic instabilities that may augment tumorigenesis [100], Kcr-mediated DNA damage repair [32] and accurate spindle positioning [33] are conducive to maintaining genomic integrity and blocking tumour development.

### Lactylation

#### Characteristics and functions of Kla

Lactate-derived Kla is a newly identified PTM that closely couples metabolism, especially the ‘Warburg effect’, with epigenetic modification. Under lactate increases induced by hypoxia and bacterial challenges, histone Kla is stimulated and directly promotes gene transcription [21]. A recent study showed that lactyl-CoA, not lactate or (R)-S-lactoylglutathione, demonstrates significant and specific binding to P300 to mediate histone Kla [101]. In addition, protein Kla is realized in a nonenzymatically modulated manner and has been comprehensively described. In protein Kla, the acyl group is derived from the secondary glycolytic intermediate lactoylglutathione (LGSH), and elevated LGSH indicates an increase in Kla-modified glycolytic enzymes and significantly represses glycolytic output. Therefore, the role of Kla in glycolysis can be interpreted as a feedback mechanism in which hyperglycaemic conditions promote LGSH generation and Kla, thus suppressing glycolytic flux and cell growth [102].

The levels of acetyl-CoA and lactate are increased by Gli-like transcription factor 1 (Glis1), which directly binds to and opens chromatin at glycolytic genes while closing chromatin at somatic genes. Therefore, H3K27ac and H3K18la on the promoters of pluripotency genes have been shown to activate their expression and induce pluripotency acquisition, forming an epigenome–metabolome–epigenome signalling cascade for cell reprogramming [39]. Because elevated histone Kla induced by lactate production is located on the promoters of profibrotic genes in alveolar macrophages, lactate along with histone Kla may be an unconventional and putative target to treat lung fibrosis [40]. In addition, in the late phase of M1 (proinflammatory) macrophage polarization, increased histone Kla induces homeostatic gene expression and facilitates the acquisition of the M2 (anti-inflammatory)-like phenotype, which may inhibit macrophage activation, dampen the inflammatory response and prevent further tissue damage [21]. This transition also occurs in colitis dictated by a Toll-like receptors (TLRs) signalling adapter via histone Kla [41].

#### Role of Kla in cancer

More recently, pioneering work has provided the first compelling insight into the role of histone Kla in ocular melanoma. H3K18la promotes the expression of YTHDF2, which recognizes N^6^-methyladenosine (m6A)-modified PER1 and TP53 mRNAs and enhances their degradation, thus driving oncogenesis [103]. Considering the finding that increased histone Kla in the late phase of M1 macrophage polarization drives an M2-like phenotype, which is characteristic of tumour-associated macrophages (TAMs) [21, 101], an indirect role for Kla in cancer growth and proliferation has been proposed, and histone Kla may become a potential tumour therapy target. Furthermore, because lactate is an oncometabolite produced by the ‘Warburg effect’ and a global epigenetic regulator, lactate-induced Kla may be involved in tumorigenesis [21]. For example, a study established that lactate promotes cell proliferation and modulates cellular metabolism at least in part through histone lactylation-mediated gene expression in non-small cell lung cancer cells [104]. Additionally, the increase in lactate in breast cancer significantly boosts the transcription of tumour-related genes such as key oncogenes and cell cycle genes, which may be partially ascribed to histone Kla on relevant gene promoters. However, this scenario warrants further investigation [105].

### Succinylation

#### Characteristics and functions of Ksucc

Ksucc, the covalent deposition of a succinyl group on the ε-amino group of lysine, was first identified and verified in *Escherichia coli* and can induce significant chemical changes in proteins to promote important cellular functions [22]. Histone Ksucc marks are distributed widely in HeLa cells, mouse embryonic fibroblasts (MEFs), Drosophila S2 cells, and *Saccharomyces cerevisiae* cells, showing that histone Ksucc is highly conserved among species. H2AK95, H2BK116, H2AK95 and H2BK120 are unique Ksucc sites that have rarely been reported for other PTMs, most of which occur in the globular domain (GD) and the C-terminus of histones, where they are more accessible for participation in chemical reactions [106]. Nonenzymatic Ksucc is mediated by succinyl-CoA, which serves as the entry point of odd-numbered fatty acids and branched-chain amino acids into the tricarboxylic acid (TCA) cycle [107, 108], while enzymatic Ksucc can also be mediated by succinyltransferases, such as the α-ketoglutarate dehydrogenase complex (KGDHC) [109], GCN5 [110–112], histone acetyltransferase 1 (HAT1) [113] and carnitine palmitoyltransferase 1A (CPT1A) [114–116], and desuccinylases, such as SIRT5 [117, 118] and SIRT7 [119]. The GAS41 YEATS domain is the ‘reader’ of the Ksucc mark on H3K122 [120].

Hypersuccinylation in chromatin is caused by succinyl-CoA accumulation and results in enhanced transcriptional responses. In other words, histone Ksucc is an inextricable link between the TCA cycle status and epigenetic transcriptional control [42]. Truly, histone Ksucc can impinge upon nucleosome dynamics and foster DNA unwrapping from the histone surface, thus allowing proteins, such as transcription factors, to easily access buried regions of nucleosomal DNA [43]. In view of the prominent role of succinyl-CoA in the TCA cycle, relevant Ksucc-modified proteins play roles in numerous metabolic pathways, including the oxoacid metabolism, purine/pyrimidine metabolism, glycolysis/gluconeogenesis, and pyruvate metabolism pathways, in many species [108, 118, 121–125]. Additionally, Ksucc of core mitochondrial enzymes undermines their stability and activity, thus impairing mitochondrial respiration, mitophagy and metabolic flexibility [44]. In congenital metabolic disorders with succinyl-CoA ligase (SCL) deficiency [126], global protein hypersuccinylation caused by succinyl-CoA accumulation may partially contribute to hereditary mitochondrial encephalomyopathy [127]. Ksucc is also involved in solubilizing amyloid plaques and tangles and deterring the aggregation of neurofilaments [45–48], consistent with its notable decline in the Alzheimer’s disease (AD) brain [128–131]. In cardiovascular diseases, hypersuccinylation of ECHA [i.e., caused by SIRT5 knockout (KO)] impairs its activity and ATP production to cause hypertrophic cardiomyopathy and increased mortality under chronic pressure overload [49, 50]. Moreover, augmented Ksucc of succinate dehydrogenase (SDH) boosts its activity and exacerbates ischaemia-reperfusion injury in SIRT5-KO mouse hearts [51–53]. Additionally, Ksucc plays an active role in drug resistance [121], fungal pathogenicity [132], hypothyroxinemia [133], colitis [134] and other pathological activities.

#### Role of Ksucc in cancer

A large number of investigations have revealed the role of Ksucc as a double-edged sword in tumorigenesis. Researchers have found that Ksucc exerts a positive effect on tumorigenesis through histone and nonhistone modifications. In human pancreatic ductal adenocarcinoma (PDAC), GCN5 catalyses the H3K79succ modification on the promoter region of YWHAZ to promote its expression, which reinforces the proliferation, migration and invasion of PDAC cells [111]. This GCN5-mediated H3K79succ modification is also involved in liver cancer [112] and glioblastoma [110] formation. Furthermore, Ksucc profoundly modifies nonhistone proteins and fosters tumorigenesis. In gastric cancer (GC), Ksucc of S100A10 and lactate dehydrogenase A (LDHA) is significantly increased in GC with inhibited degradation, enhancing tumour invasion and migration [115, 116], while desuccinylation of 2-oxoglutarate dehydrogenase (OGDH) by SIRT5 dampens its activity and subsequently suppresses tumorigenesis in GC [135]. K569succ of caldesmon (CALD1), which is closely related to tumour metastasis, is profoundly decreased in GC and may be a viable biomarker for GC [136]. In addition, K311succ of GLS in PDAC [137], K118succ of LDHA in prostate cancer [138], K123succ of Cu/Zn superoxide dismutase (SOD1) in lung cancer [139], K433succ of pyruvate kinase M2 (PKM2) in colon cancer [140], Ksucc of H3K122 and phosphoglyceromutase 1 (PGAM1) catalysed by HAT1 [113] and hypersuccinylation induced by R-2-hydroxyglutarate [141] all have a far-reaching and positive impact on tumorigenesis.

However, emerging evidence also suggests that SIRT5-mediated desuccinylation could potentiate tumorigenesis by inactivating succinate dehydrogenase complex subunit A (SDHA) in renal cell carcinoma [142], stabilizing GLS in breast cancer [13, 143], activating citrate synthase (CS) and serine hydroxymethyltransferase (HMT2) in colon cancer [144, 145] and inhibiting PKM2 in lung cancer [146]. These studies collectively suggest an avenue for targeting SIRT5 in a tumour therapeutic strategy executed via pharmacological inhibition [147]. In addition, hyposuccinylation induced by reduced succinyl-CoA levels has been demonstrated in oesophageal squamous cell cancer (ESCC) and may alter ESCC metabolism and promote cell migration [148]. Similarly, in renal cell carcinoma, Ksucc has also been found to be connected with tumour energy metabolism [149]. Moreover, in colon cancer cells treated with dichloroacetate (DCA), a glycolysis inhibitor, elevated proteins labelled with Ksucc may have important functions in turning the ‘Warburg effect’ [150, 151] into normal oxidative phosphorylation to mediate the antitumour effect of DCA [152].

Collectively, these studies show that the function of Ksucc in tumour development is context-dependent and that the regulatory pathway that is predominant may be critical in determining whether Ksucc functions as a tumour suppressor or tumour promoter.

### Propionylation and butyrylation

#### Characteristics and functions of Kpr and Kbu

Kpr and Kbu have been concurrently identified on histones in mammalian cells, as well as in yeast core histones, indicating the evolutionary conservation of these modifications in eukaryotic cells [23, 153, 154]. Propionyl-CoA, obtained through the catabolism of odd-numbered fatty acids and branched-chain amino acids, is the putative substrate for Kpr, whereas butyryl-CoA derived from even-chain fatty acids provides a butyryl group for Kbu [23, 155]. P300, CREB-binding protein (CBP) and HBO1 can catalyse Kpr and Kbu as ‘writers’ [23, 73, 156]. Kpr can also be mediated by the GNAT superfamily (GCN5 and PCAF) and MYST family (MOF, MOZ and HB01) [157, 158]. SIRT1–3, histone deacetylases, can remove the propionyl and butyryl groups from lysine residues in the presence of NAD+ [153, 156, 159]. As a ‘reader’ of Kpr and Kbu, the bromodomain of BRD4 can bind these acyl groups with binding affinities that are significantly lower than its binding affinity for Kac [160], while the YEATS domain has enhanced affinities for Kpr and Kbu over Kac [80]. The bromodomains of BRD9 and CECR2 and the second bromodomain of TAF1 also recognize the longer butyryl mark and thus promote protein modification [84].

Histone Kpr and Kbu can be reversibly modulated upon metabolic perturbation, being inhibited upon glucose deprivation and rescued by glucose replenishment [54], providing a novel epigenetic regulatory mark of cell metabolism. Moreover, histone Kpr and Kbu stimulate transcription to an extent similar to that of histone Kac [54, 55, 57]. For instance, in spermatogenic cells, the oscillation of histone Kac and Kbu at gene TSSs may be a determinant in maintaining high transcriptional activities during spermatogenesis [58]. Researchers have identified that abnormal accumulation of these modifications at the total protein level caused by defective acyl-CoA metabolism may greatly impact protein functions and shed light on the pathophysiology of congenital metabolic disorders [60]. In addition, Kpr of bovine carbonic anhydrase II (BCA II) has been implicated in promoting amyloid fibrillation and protein aggregation, partially contributing to the formation of systemic amyloidosis and neurodegenerative diseases [56].

#### Role of Kpr and Kbu in cancer

Extensive studies provide a glimpse into the potential roles of Kpr in cancer diagnosis and therapy. The sharp reduction in Kpr of H3K23 in the U937 leukaemia cell line during monocytic differentiation implies that the initial hyperpropionylation in U937 cells might be a stage-specific marker during haematopoiesis and leukaemogenesis and may serve as a diagnostic indicator for leukaemia therapy [153]. It has also been reported that human bromodomain- and PHD finger-containing protein 1 (BRPF1) variants that are typically expressed in multiple cancers [161, 162] impair BRPF1-dependent activation of KAT6A for propionylation of H3K23, indicating the previously unrevealed therapeutic strategy of targeting the BRPF1 mutation and Kpr in cancers [74]. Histone deacetylase inhibitors (HDACis), as antitumour drugs, can induce not only histone Kac but also Kpr in glioma cells, suggesting that Kpr may be measured to monitor HDACi pharmacological actions and their influence on interactions in malignant cells [163]. Additionally, propionate, a short-chain fatty acid (SCFA) that originates from bacterial fermentation of dietary fibres in the colon [164], has been shown to induce overall Kpr, which upregulates MICA/B surface expression and suppresses the development of colon cancer [165].

Analogously, histone Kbu can be upregulated in tumour cells by HDACis, such as suberoylanilide hydroxamic acid (SAHA) in neuroblastoma [166], largazole-7 in colorectal cancer [167], and sodium butyrate (NaB) in Ewing sarcoma [168], indicating its unique influences on the antitumour activities of HDACis. Lysine butyrylated sites on core histones have been identified in ESCC cell lines, including H3K18, H3K23, H3K79 and H4K77 [169]. In addition, one of the potent delivery ligands for tumour therapy is GnRH-III[4Lys(Bu), 8Lys (Dau = Aoa)] with Kbu at position 4, which has a higher binding affinity, higher enzymatic stability, higher cellular uptake and higher tumour growth inhibitory activity than GnRH-III bioconjugates with Kac [170, 171].

### Malonylation

#### Characteristics and functions of Kma

Kma was first identified as an evolutionarily conserved PTM in prokaryotes and eukaryotes in 2011 [24]. In 2012, scientists found malonyllysine sites in histones of HeLa and *S. cerevisiae* cells [106]. Several histone Kma marks have been found in C-terminal GDs, signifying the distinction between the roles of Kma and Kac in cellular regulation [61]. Considering that the citrate-derived metabolite malonyl-CoA is the precursor of de novo fatty acid synthesis and a critical inhibitor of fatty acid oxidation [172] and because it supplies substrates for Kma [24], the elevation of Kma upon inhibited fatty acid synthase activity may be ascribed to the accumulation of malonyl-CoA [173]. SIRT5 catalyses lysine demalonylation reactions in human cells [24, 117], while SIRT2, as a yeast sirtuin homologue, might remove this modification in budding yeast or fission yeast [174].

In diabetes-induced neural tube defects (NTDs), histone Kma is elevated both in vitro and in vivo, offering new insights into the pathological effect of histone Kma in human NTDs [62]. In addition, global profiling of proteins modified by Kma in *E. coli* demonstrated that this modification was intimately associated with energy metabolism, especially with fatty acid synthase and the TCA cycle [173]. Moreover, hypermalonylation in SIRT5-KO mice modified and suppressed glyceraldehyde-3-phosphate dehydrogenase (GAPDH) to redirect glucose away from oxidation towards glycogen synthesis or the pentose phosphate pathway (PPP) in primary hepatocytes [175]. In lipopolysaccharide (LPS)-stimulated macrophages, increasing malonyl-CoA induced increased Kma levels at the 213 site of GAPDH, which spurred proinflammatory cytokine production by modulating GAPDH activity and mRNA-binding capacity, which are inflammatory signals in macrophages [59]. In malonyl-CoA decarboxylase (MCD)-deficient disease, increased Kma due to malonyl-CoA accumulation impairs mitochondrial function and fatty acid oxidation, suggesting that Kma plays a role in the pathophysiology of malonic aciduria [60, 61]. In addition, the Sirt5-Kma pathway may contribute to altered chondrocyte metabolism during osteoarthritis development since SIRT5-deficient chondrocytes exhibit increased Kma levels and decreased glycolysis and mitochondrial respiration rates [63].

#### Role of Kma in cancer

SIRT5 can not only demalonylate and inactivate SDHA to cause multidrug resistance in wild-type Kras colorectal carcinomas (CRCs) [176] but also mediate TPI demalonylation and impair its formation and activity, causing recurrence of mutant Kras CRC [177]. These findings may lead to new possibilities for achieving long-term CRC remission through suppression of SIRT5-mediated demalonylation in combination with chemotherapy. Moreover, by elevating malonyl-CoA levels, fatty acid synthase inhibition has been implicated in the promotion of mTOR lysine malonylation, which impairs angiogenesis, underscoring the importance of mTOR Kma in tumorigenesis since it may be an effective weapon against pathological neovascularization [178].

### Glutarylation

#### Characteristics and functions of Kglu

Pioneering work led to the first report of Kglu in 2014. Kglu modifies metabolic enzymes and mitochondrial proteins [25]. Kglu can be nonenzymatically driven with glutaryl-CoA, which is an important intermediate in lysine and tryptophan metabolism [25]. The removal of a glutaryl group is known to be primarily performed by SIRT5 and SIRT7 in an NAD^+^-dependent manner, while P300 and GCN5 are known as ‘writers’ of Kglu [25, 64].

Of particular interest, the core histone H2B has been revealed to have three Kglu sites (H2BK5, H2BK116, and H2BK120), which may be critical for chromatin-mediated processes, such as the regulation of gene expression [25]. Indeed, H4K91glu can destabilize nucleosome structure by interrupting histone-histone interactions to create a more relaxed environment for DNA accessibility and gene transcription [64]. A feedback loop in which glutaryl-CoA is produced within the lysine/tryptophan oxidation pathway impedes GCDH function via glutaryl modification and downregulates lysine catabolism [14]. Mutations in the GCDH gene cause the neurometabolic disorder glutaric aciduria type 1 (GA1), featuring protein hyperglutarylation, which impairs enzymatic activity and protein interactions to disrupt mitochondrial heterogeneity [65]. Protein Kglu has also been revealed to maintain sperm motility and may be involved in the aetiology of asthenozoospermia [66].

#### Role of Kglu in cancer

SIRT5 overexpression is significantly correlated with poor prognosis in CRC, and scientists have found that glutamate dehydrogenase 1 (GLUD1) can be deglutarylated and activated via SIRT5 to promote cellular glutaminolysis, thus enhancing colorectal carcinogenesis. This finding suggests that SIRT5 and Kglu are promising targets for the selective killing of cancer cells [179]. However, another study revealed that SIRT5 protects cells from oxidative damage via isocitrate dehydrogenase 2 (IDH2) desuccinylation and glucose-6-phosphate dehydrogenase (G6PD) deglutarylation, and SIRT5-KO upregulates cellular susceptibility to oxidative stress [180], which is vital to tumorigenesis [181, 182]. The abovementioned evidence shows the complexity of SIRT5 and Kglu regulation of tumour progression.

### 2-Hydroxyisobutyrylation

#### Characteristics and functions of Khib

Khib has been found to be widely distributed on both histone and nonhistone proteins in HeLa cells, MEFs, Drosophila S2 cells, yeast (*S. cerevisiae*) cells and developing rice seeds [26, 183], verifying its evolutionarily conserved and dynamic role among many species. Among 63 human and mouse histone Khib sites, nearly one-half are neither Kac sites nor Kcr sites [26], uncovering the discriminative effects of Khib on cellular modulation. Khib is likely derived from 2-hydroxyisobutyrate donors, one of which may be 2-hydroxyisobutyryl-CoA (Hib-CoA). The Khib pathway reprogrammes epigenetic networks in response to dynamic changes in the cellular metabolite Hib-CoA [26]. The acetyltransferase Esa1p in budding yeast and its homologue Tip60 in humans as well as P300 can add a 2-hydroxyisobutyryl group to substrate proteins [184, 185], whereas demodification can be achieved by HDAC1–3 [26], Rpd3p (class I HDAC), Hos3p (class II HDAC) [186] and CobB [187].

H4K8hib is enriched at the TSSs of genes and is associated with active gene transcription in meiotic and postmeiotic cells [26]. H4K8hib is a dynamic modification that orchestrates glucose level changes within cells and establishes a link between histone modification and carbon metabolism [186].

#### Role of Khib in cancer

HDAC3-mediated de-2-hydroxyisobutyrylation of H4K8 on the covalently closed circular DNA (cccDNA) minichromosome of hepatitis B virus (HBV) can be enhanced by interferon-α to inhibit HBV transcription and replication in hepatoma cells [188], which may hinder liver cancer formation. Quantitative proteome studies have identified core histones modified by Khib, Kbu, Kpr, and Ksucc in HSP90 inhibitor-treated bladder cancer cells, and the results indicated an affinity between epigenetic modifications and HSP90 inhibitor-mediated antitumour effects [189]. Since oncomutations of linker histones (LHs; H1/H5) occur mainly near PTM sites and because the most common site in the GD of histone H1.2 is modified by Khib, these mutations are thought to potentially block the primary PTM sites and interfere with their reading, writing and/or erasing processes, thus regulating gene expression in cancer cells [190]. Moreover, the reduction in the K281hib level on ENO1 induced by aspirin leads to inhibited ENO1 activity and is critical for attenuating glycolysis and the proliferation of hepatoma cells [191]. Protein expression within the actin cytoskeleton regulatory pathway and Khib modification levels are significantly altered in oral squamous cell carcinoma, which may indicate their importance for tumour progression [192]. Additionally, Khib-modified proteins are abundant in carbohydrate metabolism pathways, especially metabolic pathways in cancer [193].

### β-Hydroxybutyrylation

#### Characteristics and functions of Kbhb

Kbhb, a widespread histone mark in human and mouse cells, can be dramatically induced under prolonged fasting conditions and diabetic ketoacidosis [27] because an elevated ketone body, β-hydroxybutyrate (BHB), is a substrate in the generation of β-hydroxybutyryl-CoA that can induce P300-mediated histone Kbhb [194, 195]. In contrast to the unspecialized Zn-dependent HDAC1–3 removal of the β-hydroxybutyryl group [196], human SIRT3 displays class-selective histone de-β-hydroxybutyrylase activities favouring H3K4, H3K9, H3K18, H3K23, H3K27, and H4K16, but not H4K5, H4K8, or H4K12, suggesting a potential regulatory mechanism involving hierarchical gene repression under metabolic alterations [197]. However, treatment with NaBut, 4-PBA or SAHA, all of which are classical HDACis, can promote histone Kbhb to an even greater extent than BHB [194].

Kbhb is abundant in the GD of the LHs H1.4 and H1.5, influencing the interaction of LHs with both DNA and the nucleosome, similar to Khib [190]. Because Kbhb, especially H3K9bhb, elevates and upregulates gene expression in starvation-responsive metabolic pathways in the mouse liver during prolonged fasting, a feedback mechanism is obviously formed; thus, metabolite production altered by cellular energy conditions can influence histone Kbhb and relevant gene expression to maintain homeostasis [27]. In addition, ketogenesis proceeds through an unusual metabolic pathway that links the epigenetic modification required for memory development of CD8+ T memory cells by prompting BHB production and deposition of H3K9bhb marks on relevant genes [67]. In obese diabetic mice treated with dapagliflozin, a selective competitive inhibitor of sodium/glucose cotransporter 2 (SGLT2), elevated β-hydroxybutyric acid levels mediated Kbhb of H3K9 to promote the expression of the adiponectin gene in adipocytes, partially accounting for the molecular mechanisms by which SGLT2 inhibitors protect against cardiovascular events in diabetic patients [198]. BHB-induced Kbhb of H3K9 can antagonize glomerulosclerosis induced by diabetes and alleviate depressive behaviours by modulating specific gene expression through promoters [68, 69].

#### Role of Kbhb in cancer

Metastasis-associated protein 2 (MTA2) was upregulated in hepatocellular carcinoma (HCC) cells and induced the accumulation of βHB, increased the level of H3K9bhb, and exerted a cascading effect on HCC cell stemness and progression [199]. P53 encoded by the Tp53 gene acts as an essential tumour suppressor and promotes cell growth arrest and apoptosis [200, 201]. A recent study revealed that p53 can be modified by Kbhb at K120, K319, and K370 and can be inactivated in BHB-treated tumour cells. Specifically, Kbhb of p53 results in reduced p53 acetylation and expression of the downstream genes p21 and PUMA, thereby facilitating cell growth. It can also be deduced that Kbhb of P53 partially explains the role of ketone bodies in tumour biology [202]. In addition, P53 can also be modified by Ksucc, Kpr, Kbu, and Kcr, indicating that these modifications may affect P53-mediated tumour inhibition [23, 156, 203, 204].

## Conclusions and future perspectives

**Fig. 2:**
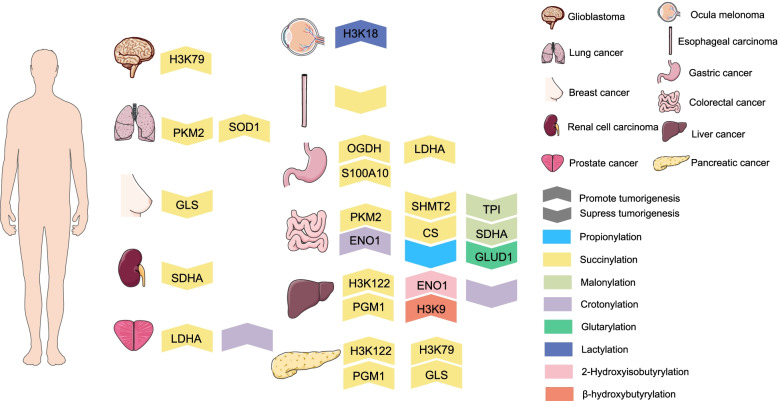
Regulatory roles of lysine acylation in tumorigenesis. Lysine acylation exerts profound effect on diverse tumour formation. In glioblastoma, H3K79succ promotes gene expression and tumour growth. In lung cancer, Ksucc of SOD1 impedes its anti-tumor effect while desuccinylation of PKM2 boost tumour growth. In breast cancer, hypersuccinylation causes GLS degradation and impedes glutamine consumption of tumour cells. In renal cell carcinoma, SDHA is desuccinylated and fosters tumour proliferation. In prostate cancer, Ksucc of LDHA increases its activity in promoting tumour metastasis and histone hypocrotonylation induced by BRD4 inhibitors hampers tumour proliferation and migration. In ocular melanoma, H3K18la drives oncogenesis. In ESCC, restoring Ksucc level restricts cell growth, migration and invasion. In GC, elevated Ksucc of S100A10 and LDHA enhances tumour invasion and migration whereas desuccinylation of OGDH suppresses tumorigenesis. In colorectal cancer, Ksucc of PKM2 and Kcr of ENO1 promotes cell survival and tumour development and desuccinylation of CS and SHMT2 accelerates colon cancer growth. Kpr induced by propionate suppresses the development of colon cancer. Demalonylation of SDHA and TPI prompts the recurrence of CRC and GLUD1 can be deglutaryled to promote colorectal carcinogenesis. In liver cancer, Ksucc of H3K122 and PGM1 are required for liver cancer growth. Repressed Khib of ENO1 causes proliferation defective of liver cancer cells and H3K9bhb participates in the promotion of HCC stemness and progression. Increasing Kcr level leads to undermined liver cancer cell migration and proliferation. In PDAC, Ksucc of H3K79, H3K122, PGM1 and GLS promotes cell proliferation, migration and invasion

A growing body of breakthrough discoveries on novel acylation types is creating new opportunities for deeper investigations into the nonmetabolic roles of metabolites in tumorigenesis. Histone acylation inextricably links the epigenome with the metabolome via transcriptional modulation, and nonhistone acylation alters protein functions to transmit proteomic-metabolomic signals (Table 2). Accordingly, despite metabolites being downstream products of manifold biological activities, metabolites have profound impacts on upstream biosystems and serve as ‘drivers’ of diverse biological processes [1]. Because acylation can be influenced by the availability of acyl-CoA through both enzymatic and nonenzymatic mechanisms [205], a shift in cellular utilization of energy sources in tumours induces aberrant acylation [206], which has been acknowledged as a general mechanism of cancer cell regulation and is highly conserved and deleterious [207]. Specifically, altered acylation affects tumour metabolism to promote tumorigenesis by influencing gene expression and cell signalling in multiple tumour types (Fig. 2). The abovementioned increase in research on acylation types that we reviewed based on acylation category directly and indirectly paves the way for a greater understanding of acylation-driven feedback loops.

**Table 2:** Role of aberrant lysine acylations in tumorigenesis

Acylation	Tumour type	Histone/protein involved	Description	Ref
Kcr	Prostate cancer	/	Hypocrotonylation on histones induced by BRD4 inhibitors hampers the proliferation and migration of prostate cancer	[32]
	Colorectal cancer	H3K18;ENO1	Intestinal microbiota depletion resultes in increased expression of HDAC2 to downregulate Kcr level and relates to tumorigenesis in colon cancer;K420cr of ENO1 facilitates the proliferation, migration and invasion of colorectal cancer	[34–36, 89]
	Leukemia	/	Efficiently epigenetic impact of YEATS domain on leukemia may partially owing to its interaction with histone Kcr	[37, 38]
	Liver cancer	/	Kcr expression correlates with TNM stage in liver cancer and increasing Kcr level leads to undermined cell migration and proliferation	[90]
	Long cancer	CAV1; Complement C3	CAV1 and Complement C3 as regulators of tumour metastasis or tumour microenvironment are significantly regulated with Kcr marks	[91]
Kla	Ocular melanoma	H3K18	H3K18la promotes the expression of YTHDF2 and enhances the degradation of m6A modified PER1 and TP53 mRNAs thus driving oncogenesis	[99]
	Lung cancer	/	Lactate promotes cell proliferation and modulates cellular metabolism at least in part through histone lactylation-mediated gene expression in non-small cell lung cancer cells	[100]
	Breast cancer	/	Regulated transcription of key oncogenes, tumour suppressors as well as cell cycle and proliferation genes may be partially ascribed to histone Kla on relevant gene promoters in breast cancer	[101]
AM	Human pancreatic ductal adenocarcinoma	H3K79; H3K122;PGAM1;GLS	H3K79succ promotes YWHAZ expression and represses β-catenin degradation to enhance cell proliferation, migration and invasion of PDAC; Ksucc of H3K122 and PGM1 mediated by HAT1 are required for pancreatic cancer growth; Ksucc of GLS facilitates its oligomerization and activity to promote tumour growth of PDAC	[52, 104, 106]
	Liver cancer	H3K79;H3K122;PGAM1	Ksucc of H3K79 plays a vital role in HBV infection and liver tumour progression; Ksucc of H3K122 and PGM1 mediated by HAT1 are required for liver cancer growth	[105, 106]
	Glioblastoma	H3K79	H3K79succ promotes gene expression and tumour growth in glioblastoma cells	[103]
	Gastric cancer	S100A10;LDHA; CALD1;OGDH	Elevated Ksucc of S100A10 and LDHA hinders their degradation thus enhancing tumour invasion and migration; desuccinylation of OGDH dampens its activity and subsequently suppresses tumorigenesis in GC; K569succ of CALD1 significantly decreases in GC and may function as a promising biomarker	[50, 51, 108, 109]
	Prostate cancer	LDHA	Ksucc of LDHA increases its activity in promoting prostate tumour metastasis	[53]
	Lung cancer	SOD1;PKM2	K123succ of SOD1 decreases its activity in antioxidation and anti-tumor effect in lung tumour cells; desuccinylation of PKM2 impedes its activity to eliminate reactive oxygen species(ROS) and boost tumour growth	[132, 139]
	Colon cancer	PKM2;CS;SHMT2	K433succ of PKM2 induced its mitochondrial translocation to promote cell survival and tumour development against nutritional depletion; desuccinylation of CS accelerates colon cancer growth; SIRT5-mediated desuccinylation of SHMT2 increases its activity to foster tumour progression; elevated Ksucc caused by DCA may help to realize DCA’s anti-tumor effect	[133, 137, 138, 146]
	Renal cell carcinoma	SDHA	SDHA is desuccinylated and fosters tumour proliferation; Ksucc is intertwined with energy metabolism in RCC cells	[135, 142]
	Breast cancer	GLS	Hypersuccinylation brings about the degradation of GLS and impedes glutamine consumption of tumour cells	[14, 136]
	Esophageal squamous cell carcinoma	/	Ksucc is reduced in ESCC and restored Ksucc restricts cell growth, migration and invasion	[141]
Kpr	Leukemia	H3K23	Hyperpropionylation in leukemia cell may corelate with hematopoiesis and leukemogenesis	[147]
	Glioma	/	Kpr induced by HDACi might become a monitor of HDACi’s pharmacological actions and interactions with malignant cells	[57]
	Colon cancer	/	Kpr induced by propionate facilitates NKG2D ligand expression and holds promise for immune activating anticancer therapy	[60]
Kbu	Neuroblastoma	H2BK5, H4K12	Kbu induced by SAHA reconstructs chromatin and reactivates gene expression to inhibit tumorigenesis	[56]
	Colorectal cancer	H2BK5, H3K18 and H3K23	Kbu induced by largazole-7 may partially account for its antitumor effect	[161]
	Ewing sarcoma	/	Kbu induced by NaB may take part in its inhibiting tumour growth effect	[162]
	Esophageal squamous cell carcinoma	H3K18, H3K23, H3K79 and H4K77	Abundant histone sites with Kbu have been found in ESCC and may associate with tumour growth	[163]
Kma	Colorectal cancer	SDHA;TPI	Demalonylation of SDHA and TPI impairs their activities and prompts the recurrence of CRC	[172, 173]
Kglu	Colorectal cancer	GLUD1	GLUD1 can be deglutaryled at K545 and activated to promote cellular glutaminolysis and colorectal carcinogenesis	[176]
Khib	Liver cancer	H4K8;ENO1	de-2-hydroxyisobutyrylation of H4K8 on HBV cccDNA minichromosome can restrict HBV transcription and replication in hepatoma cells; K281hib of ENO1 can be repressed by aspirin to cause proliferation defective of liver cancer cells	[183, 186]
	Bladder cancer	/	Khib-modified histones involve in HSP inhibitors-treated bladder cancer cells to inhibit tumour progression	[184]
	Oral squamous cell carcinoma	/	The protein expression within the actin cytoskeleton regulatory pathway and their Khib modification levels significantly changes in oral squamous cell carcinoma, which may be important for tumour progression	[187]
Kbhb	Liver cancer	H3K9	H3K9bhb participates in the promotion of HCC stemness and progression induced by MAT2	[197]
	Multiple cancers	P53	P53 is modified by kbhb at lysines120, 319, and 370 and attenuates its anti-tumor effect in tumour cells	[68]

The potential crosstalk among lysine acylations also draws our attention since their corresponding metabolic sources are intertwined with each other. For instance, ccrotonyl-CoA is obtained from both glutaryl-CoA and butyryl-CoA, and propionyl-CoA can also be transformed into succinyl-CoA (Fig. 1). Furthermore, because regulatory enzymes, including writers, erasers and readers of acylation, overlap, these modifications are likely to change concurrently in metabolic disturbances or any other pathological state and may function together to mediate cellular signalling. For example, SIRT5 promotes IDH2 desuccinylation and G6PD deglutarylation to enhance cellular antioxidant defence [180]. It is also worth noting that acylations and other PTMs often modify the same protein such as P53, H3, LDHA and ENO1 (Fig. 2), in an agonistic or antagonistic manner. The β-hydroxybutyrylation of p53 results in reduced p53 acetylation, which affects the transcriptional activity of p53 on its target gene [202]. In addition, H2A-K119 malonylation inhibits H2A S121 phosphorylation, showing novel crosstalk between lysine malonylation and serine/threonine phosphorylation [174]. We assume that there must be more precise and specific interplay between the different acylations and other PTMs, and substantial effort will be required to map out and carefully dissect these interactions.

However, since acetylation occurs ubiquitously in diverse cellular biological processes, its important role in tumour regulation cannot be fully understood. Notably, many regulatory enzymes for the aforementioned novel acylation types were first found to catalyse acetylation or deacetylation. Therefore, the acetylation level can concomitantly change in conjunction with other acylation levels upon the modulation of enzymatic activity, which may confuse the outcomes. For example, Kac is significantly elevated in parallel to other acylation types and cannot be excluded as a cause for any of the antitumour effects of HDACis [163, 166–168]. In addition, it is essential to identify whether these novel types of acylation function synergistically and comprise a ‘nonacetyl’ group or behave independently in cellular regulation. Furthermore, because one specific acylation can modify different enzymes in various metabolic pathways or on different sites in the same protein, such as Ksucc of PKM2 [140, 146] and GLS [13, 137], the sophisticated modification paradigm may cause different, even completely opposite, results. Therefore, it is important to specify whether these acylation types form a complicated network that includes diverse proteins in a single disease and determine ways to prevent unwanted effects and strengthen desired influences in cancer therapy. In contrast to the abundant recognition of nonhistone acylation, discoveries of histone acylation remain scant. Further efforts to identify new histone sites with novel acylation types are urgently needed to expand the scope of knowledge on epigenetic alterations affecting tumour heterogeneity [208], since precise and effective personalized therapy using epidrugs has emerged [209]. After resolving these conundrums, lysine acylation will likely play a burgeoning role in tumour diagnosis, monitoring and/or treatment in the clinic.

Despite considerable efforts to understand the relevance of metabolite-derived PTMs in the cellular context, more progress is needed in the future to identify their tremendous impact, which will ultimately expand the roles of metabolites in tumorigenesis.

## Data Availability

Not applicable.

## Abbreviations

Ac-CoA: Acetyl-CoA or acetyl coenzyme A
AD: Alzheimer’s disease
α-KG: α-ketoglutarate
α-KGDH: α-ketoglutarate dehydrogenase complex
AKI: Acute kidney injury
BCA II: Bovine carbonic anhydrase II
BHB: β-hydroxybutyrate
BMDM: Bone marrow-derived macrophage
BRPF1: Bromodomain- and PHD finger-containing protein 1
BRD4: Bromodomain-containing protein 4
CALD1: Caldesmon
CBP: CREB-binding protein
cccDNA: Covalently closed circular DNA
CDYL: Chromodomain Y-like protein
CPT1A: Carnitine palmitoyltransferase 1A
CRC: Colorectal carcinomas
CS: Citrate synthase
DCA: Dichloroacetate
DPF: Double PHD finger
DPF2: Also known as BAF45d
DSB: Double-strand break
ENO1: Enolase 1
ESCC: Oesophageal squamous cell cancer
FASN: Fatty acid synthase
GA1: Glutaric aciduria type 1
GAPDH: Glyceraldehyde-3-phosphate dehydrogenase
GC: Gastric cancer
GCDH: Glutaryl-CoA dehydrogenase
GCN5: General control of amino acid synthesis 5-like 2, also known as KAT2A
GD: Globular domain
Glis1: Gli-like transcription factor 1
GLS: Glutaminase
GLUD1: Glutamate dehydrogenase 1
G6PD: Glucose-6-phosphate dehydrogenase
HAT1: Histone acetyltransferase 1
HBO1: Also known as KAT7 or MYST2
HBV: Hepatitis B virus
HCC: Hepatocellular carcinoma
HDACs: Histone deacetylases
HDACis: Histone deacetylase inhibitors
HDR: Homology directed repair
2-HG: 2-hydroxyglutarate
Hib-CoA: 2-hydroxyisobutyryl-CoA
H3K122: Histone H3 lysine 122
H3K18: Histone H3 lysine 18
H3K23: Histone H3 lysine 23
H3K27: Histone H3 lysine 27
H3K4: Histone H3 lysine 4
H3K79: Histone H3 lysine 79
H3K9: Histone H3 lysine 9
H4K8: Histone H4 lysine 8
H4K12: Histone H4 lysine 12
H4K77: Histone H4 lysine 77
IDH2: Isocitrate dehydrogenase 2
IFN-α: Interferon-α
K164: Lysine residue 164
Kac: Lysine acetylation
KAT6A: Lysine acetyltransferase 6A
KATs: Lysine acetylatransferases
Kbu: Butyrylation
Kcr: Lysine crotonylation
Kbhb: Lysine β-hydroxybutyrylation
KGDHC: α-ketoglutarate dehydrogenase complex
Kglu: Lysine glutarylation
Khib: Lysine 2-hydroxyisobutyrylation
Kla: Lysine lactylation
Kma: Lysine malonylation
KO: Knockout
Kpr: Lysine propionylation
Ksucc: Lysine succinylation
LDHA: Lactate dehydrogenase A
LGSH: Lactoylglutathione
LHs: Linker histones
LPS: Lipopolysaccharide
m6A: N^6^-methyladenosine
MCD: Malonyl-CoA decarboxylase
MEF: Mouse embryonic fibroblasts
MOF: Also known as KAT8
MOZ: Also known as KAT6A
MSCI: Meiotic sex chromosome inactivation
MTA2: Metastasis-associated protein 2
NaB: Sodium butyrate
NAD +: Nicotinamide adenine dinucleotide
NTD: Neural tube defects
OGDH: 2-oxoglutarate dehydrogenase
PCAF: p300/CBP associated factor
PDAC: pancreatic ductal adenocarcinoma
PGAM1: Phosphoglyceromutase 1
PKM2: Pyruvate kinase isozyme M2
PPP: Pentose phosphate pathway
PTMs: Post-translational modifications
ROS: Reactive oxygen species
SAHA: Suberoylanilide hydroxamic acid
SCFA: Short-chain fatty acid
SCL: Succinyl-CoA ligase
SDH: Succinate dehydrogenase
SDHA: Succinate dehydrogenase complex, subunit A
SGLT2: Sodium/glucose cotransporter 2
SHMT2: Serine hydroxymethyltransferases
SIRT: Sirtuin
SOD1: Cu/Zn superoxide dismutase
SUCNR1: Succinate receptor 1
TAMs: Tumor-associated macrophages
TCA: Tricarboxylic acid
TIP60: Tat-interacting protein, also known as histone acetyltransferase KAT5
TLR: Toll-Like receptors
TSSs: Transcriptional start sites
